# Safety and Efficacy of Subcutaneous Pasireotide in Patients With Cushing's Disease: Results From an Open-Label, Multicenter, Single-Arm, Multinational, Expanded-Access Study

**DOI:** 10.3389/fendo.2019.00436

**Published:** 2019-07-16

**Authors:** Maria Fleseriu, Chioma Iweha, Luiz Salgado, Tania Longo Mazzuco, Federico Campigotto, Ricardo Maamari, Padiporn Limumpornpetch

**Affiliations:** ^1^Departments of Medicine and Neurological Surgery, Northwest Pituitary Center, Oregon Health and Science University, Portland, OR, United States; ^2^Panda Medical Associates, Peoria, AZ, United States; ^3^General Internal Medicine Service, Hospital das Clinicas da Faculdade de Medicina FMUSP, São Paulo, Brazil; ^4^Division of Endocrinology of Medical Clinical Department, University Hospital, UEL, Londrina, Brazil; ^5^Novartis Pharmaceuticals Corporation, East Hanover, NJ, United States; ^6^Division of Endocrinology and Metabolism, Department of Internal Medicine, Faculty of Medicine, Prince of Songkla University, Hat Yai, Thailand

**Keywords:** Cushing's disease, pasireotide sc, somatostatin analog, clinical practice, real world

## Abstract

**Introduction:** The efficacy and safety of subcutaneous (sc) pasireotide have been evaluated in a Phase III trial. Here, we report safety and efficacy results from a multinational, expanded-access study of pasireotide sc in patients with Cushing's disease (CD) in a real-world setting (clinicaltrials.gov, identifier: NCT01582061).

**Methods:** Adults with active CD previously untreated with pasireotide were enrolled; pasireotide sc was initiated at 600 μg twice daily (bid; EU countries) or 900 μg bid (non-EU countries; 600 μg bid in patients with impaired glucose metabolism). Pasireotide dose could be adjusted in 300 μg increments/decrements to a maximum of 900 μg bid or minimum of 300 μg bid for sustained urinary free cortisol (UFC) normalization/tolerability issues. Primary objective: document the safety of pasireotide sc in patients with CD. Key secondary objectives: assess the proportion of patients with mean UFC (mUFC) not exceeding the upper limit of normal (ULN) and changes from baseline in clinical signs/symptoms and quality of life (QoL) to weeks 12, 24, and 48.

**Results:** One hundred and four patients received pasireotide: female, *n* = 84 (80.8%); median duration of pasireotide exposure, 25.1 weeks; median (range) baseline mUFC, 321.2 nmol/24 h (142–10,920; 2.3 × ULN [1.0–79.2]). Forty (38.5%) patients completed the study. The most common reasons for premature discontinuation of pasireotide were unsatisfactory therapeutic effect (*n* = 26, 25.0%) and adverse events (AEs; *n* = 20, 19.2%). Drug-related grade 3/4 AEs or drug-related serious AEs (primary endpoint) were documented in 42 (40.4%) patients, most commonly diabetes mellitus (*n* = 12, 11.5%) and hyperglycemia (*n* = 8, 7.7%). All patients experienced ≥1 AE and most (*n* = 102; 98.1%) reported ≥1 drug-related AE; six (5.8%) patients discontinued treatment because of hyperglycemia-related AEs. At weeks 12, 24, and 48, respectively, 36/66 (54.5%), 22/46 (47.8%), and 9/21 (42.9%) evaluable patients had normalized mUFC levels. Clinical signs/symptoms and QoL were also improved.

**Conclusions:** In an international, real-world, clinical-practice setting, pasireotide sc was generally well-tolerated (no new safety signals were identified), effectively reduced UFC (normalization in ~50% of evaluable patients) and improved clinical signs and QoL in patients with CD. While hyperglycemia-related AEs were common, consistent with previous studies, most were manageable, with <6% of patients discontinuing treatment because of these events.

## Introduction

Cushing's disease is a rare and serious endocrine disorder characterized by a pituitary tumor (corticotropinoma) that secretes an excess of adrenocorticotropic hormone (ACTH), with consequent overproduction of cortisol from the adrenal glands (1). Sustained hypercortisolism is associated with significant morbidity, which includes, but is not restricted to, central obesity, diabetes mellitus, dyslipidemia and hypertension; prolonged exposure to elevated cortisol levels, and the associated comorbidities, may also cause impairment of quality of life (QoL) and premature mortality (2–4).

The goals of treatment are to normalize cortisol levels (5), with suppression of hypercortisolism shown to reverse signs and symptoms, alleviate the burden of morbidity, and reduce mortality (1, 4, 6–8). Surgical removal of the pituitary tumor is the first-line treatment for most patients with Cushing's disease (5). However, patients may fail to respond or experience disease recurrence (9, 10). A pooled analysis of a series of studies showed mean remission and recurrence rates after surgery of 82 and 12%, respectively, for patients with a microadenoma and 62 and 19% for patients with a macroadenoma (8). As such, effective alternative treatment strategies are required. Bilateral adrenalectomy can result in complete resolution of hypercortisolism, but it renders patients glucocorticoid and mineralocorticoid deficient and may also lead to corticotroph tumor progression (Nelson's syndrome), which is characterized by pituitary tumor expansion coupled with high ACTH concentrations (11). Radiotherapy can also be efficacious in some patients, but maximum clinical benefit is typically only achieved years after treatment initiation (11). Current medical therapies used for the treatment of Cushing's disease include adrenal-directed agents such as ketoconazole, metyrapone (both approved in the EU) and mitotane, the glucocorticoid receptor antagonist mifepristone (approved in the USA for the management of hyperglycemia associated with Cushing's syndrome), and the pituitary-targeted drugs cabergoline and pasireotide, of which the latter (twice-daily [bid] subcutaneous [sc] formulation) is approved in both the USA and the EU (5, 12). Recently, a long-acting formulation of pasireotide suitable for once-monthly administration has been approved in the EU, Japan, Canada, and the USA.

Pasireotide is a multireceptor-targeted somatostatin analog that binds to four of the five somatostatin receptor subtypes, with the highest affinity for subtype 5 (13–15). In a large Phase III study in patients with Cushing's disease (*N* = 162), pasireotide sc bid induced rapid and sustained reductions in mean 24 h urinary free cortisol (mUFC) levels, with patients also benefiting from improvements in signs and symptoms of Cushing's disease and QoL (16). Pasireotide sc was generally well-tolerated; although hyperglycemia-related adverse events (AEs) were reported in 73% of patients, only 6% of patients had pasireotide withdrawn because of these events.

While the efficacy and safety of pasireotide sc have been established in randomized, prospective studies, few studies have evaluated pasireotide sc in patients with Cushing's disease in real-world clinical practice (17–20). Here, we describe safety and efficacy results of an international, real-world study of pasireotide sc in a large population (*N* = 104) of patients with Cushing's disease in clinical practice.

## Methods

### Patients

Adult patients (≥18 years old) with persistent or recurrent Cushing's disease, or *de novo* patients not considered candidates for surgery, were recruited. Patients must have had active disease, as evidenced by: mean of three 24 h UFC samples collected during a 3-week screening period above the upper limit of normal (ULN; 137.95 nmol/24 h), which was determined from a central laboratory reference; morning plasma ACTH within or above the normal range; and confirmed pituitary source of the disease. For patients on previous medical treatment for Cushing's disease, the following washout periods were required prior to screening assessments: mitotane, 6 months; long-acting octreotide, lanreotide Autogel, 8 weeks; dopamine agonists (bromocriptine, cabergoline), mifepristone, lanreotide sustained release, 4 weeks; steroidogenesis inhibitors (ketoconazole, metyrapone, rosiglitazone), octreotide immediate release, 1 week.

Patients were excluded from the study if they had any of the following criteria: prior exposure to pasireotide sc; radiotherapy <4 weeks before screening; tumor compressing the optic chiasm, causing visual field defects; symptomatic cholelithiasis; diabetes with poorly controlled blood glucose levels (glycated hemoglobin [HbA_1c_] >8%); QTcF >450 ms at screening and any other clinically significant impairment of cardiovascular function; pregnancy.

### Study Design

This was an open-label, single-arm, multicenter, expanded-access study.

After a 21-day screening period, enrolled patients in EU countries received pasireotide sc starting doses of 900 μg bid; the study protocol was amended in 2013 (patient enrollment began in 2011) so that all EU patients received starting doses of 600 μg bid to align with the recommendation by the Committee for Medicinal Products for Human Use and the European Medicines Agency that the starting dose of pasireotide sc should be 600 μg bid (21). Patients in non-EU countries received starting doses of 900 μg bid (600 μg bid in patients with impaired glucose metabolism). The dose could be increased (after >2 months' treatment if UFC was not controlled) or decreased (for sustained UFC normalization/tolerability issues) in 300 μg increments or decrements to a maximum of 900 μg bid or a minimum of 300 μg bid.

Patients received treatment until pasireotide sc was approved for commercial use and reimbursed in each respective country or until December 31, 2015 (December 31, 2016 for sites in South Korea and Brazil), whichever occurred first.

### Objectives and Assessments

The primary objective of the study was to document the safety of pasireotide sc; the primary endpoint was the proportion of patients with drug-related grade 3/4 AEs or drug-related serious AEs (SAEs). Key secondary endpoints, which were assessed at weeks 12, 24, and 48, included: proportion of patients with mUFC ≤ULN; proportion of patients achieving at least 50% reduction from baseline in mUFC; changes from baseline in clinical signs and symptoms; and changes from baseline in health-related QoL (HRQoL).

Monitoring of safety throughout the study was carried out by performing standard safety assessments (including laboratory, vital signs, and electrocardiography evaluations) for patients with Cushing's disease and the recording of AEs at each visit. For the primary endpoint, the severity of AEs was defined as mild (grade 1), moderate (grade 2), severe (grade 3), and life threatening or debilitating (grade 4) according to the National Cancer Institute's Common Terminology Criteria for Adverse Events (CTCAE) version 3.0 (22). If CTCAE grading did not exist for an AE, the severity of mild, moderate, severe, and life threatening, corresponding to grades 1–4, was used. CTCAE grade 5 (death) was not used in this study; information about deaths was collected through a cause-of-death form. SAEs were defined as one of the following: fatal or life threatening; resulting in persistent or significant disability/incapacity; constituting a congenital anomaly/birth defect; medically significant (defined as an event that jeopardizes the patient or may require medical or surgical intervention to prevent one of the aforementioned outcomes); requiring or prolonging hospitalization.

Measurement of mUFC was determined at 4-weekly intervals until week 24; from week 24 onwards, patients were assessed at 12-week intervals. mUFC was calculated from three 24 h UFC measurements (collected during the week before visits) at weeks 12, 24, and 48 and from two 24 h UFC measurements (collected on two consecutive days before visits) at weeks 4, 8, 16, and 20. Clinical signs and symptoms, including sitting systolic and diastolic blood pressure (BP), weight, waist circumference, facial rubor, striae, bruising, hirsutism, muscle strength, and supraclavicular and dorsal fat pads, were assessed at every study visit. Facial rubor, striae, bruising, muscle strength, and supraclavicular and dorsal fat pads were assessed by the investigator; hirsutism (females only) was assessed by the Ferriman–Gallway score. Patients' QoL was assessed by the patients at weeks 4, 12, 24, 36, and 48 using a standardized 12-item Cushing's syndrome HRQoL questionnaire (CushingQoL) (23).

### Statistical Analyses

Demographics and other baseline characteristics are summarized descriptively. Categorical data are presented as frequencies and percentages. For continuous data, mean and standard deviation (SD) or median and range are described. Safety assessments were performed on all patients who received at least one dose of pasireotide sc and had at least one post-baseline safety assessment and are summarized descriptively. Treatment-emergent AEs include only AEs that started or worsened during the on-treatment period up to 28 days after discontinuation of the study drug.

Secondary efficacy assessments were performed on all patients who received at least one dose of pasireotide sc and are summarized descriptively with corresponding two-sided 95% exact confidence intervals (CIs) for weeks 12, 24, and 48. Patients were divided into two groups based on mean actual daily dose: 600 μg bid, all patients whose mean daily dose during the study was <1,500 μg/day; 900 μg bid, all patients whose mean daily dose was ≥1,500 μg/day during the study.

## Results

### Baseline Demographics and Characteristics

One hundred and four eligible patients (600 μg bid, *n* = 49; 900 μg bid, *n* = 55) from 81 centers in 12 countries received pasireotide sc between August 16, 2011 and January 26, 2017 (Table 1). Median (range) baseline UFC level for all patients was 321.2 nmol/24 h (142–10,920; 2.3 × ULN [1.0–79.2]). Median (range) baseline UFC level for the 600 μg group was 255.6 nmol/24 h (142–10,920; 1.9 × ULN [1.0–79.2]) and for the 900 μg cohort was 452.0 nmol/24 h (143–3,991; 3.3 × ULN [1.0–28.9]). Overall, most patients (*n* = 98; 94.2%) had at least one current medical condition at baseline; the most common (>15%) were hypertension (*n* = 62; 59.6%), hypothyroidism (*n* = 21; 20.2%), dyslipidemia (*n* = 20; 19.2%) and diabetes mellitus (*n* = 16; 15.4%). Eighty-four (80.8%) patients had received prior pituitary surgery at baseline, and 53 (51.0%) had been treated with medical therapy.

**Table 1:** Patient baseline characteristics and demographics.

	**Pasireotide 600 μg bid *N* = 49**	**Pasireotide 900 μg bid *N* = 55**	**All patients *N* = 104**
Mean age, years (SD)	45.5 (13.1)	39.9 (12.6)	42.5 (13.1)
Female, *n* (%)	37 (75.5)	47 (85.5)	84 (80.8)
Race, *n* (%)			
Caucasian	39 (79.6)	36 (65.5)	75 (72.1)
Black or African American	3 (6.1)	2 (3.6)	5 (4.8)
Asian	6 (12.2)	15 (27.3)	21 (20.2)
Other	1 (2.0)	2 (3.6)	3 (2.9)
Median time from diagnosis to first pasireotide dose, months (range)	60.3 (0.7–309.0)	34.3 (1.0–298.0)	39.7 (0.7–309.0)
Cushing's disease status, *n* (%)			
*De novo*	8 (16.3)	5 (9.1)	13 (12.5)
Persistent/recurrent	41 (83.7)	50 (90.9)	91 (87.5)
Previous pituitary surgery, *n* (%)			
Yes	38 (77.6)	46 (83.6)	84 (80.8)
No	3 (6.1)	4 (7.3)	7 (6.7)
Missing	8 (16.3)	5 (9.1)	13 (12.5)
Median time from previous surgery to first pasireotide dose, months (range)	44.8 (4.1–306.1)	30.5 (1.9–294.1)	38.1 (1.9–306.1)
Prior pituitary irradiation, *n* (%)			
Yes	12 (24.5)	15 (27.3)	27 (26.0)
No	37 (75.5)	40 (72.7)	77 (74.0)
Median time from last pituitary irradiation to first pasireotide dose, months (range)	56.9 (8.5–169.9)	29.0 (3.1–205.8)	33.3 (3.1–205.8)

In total, 64 (61.5%) patients discontinued treatment, 28/49 (57.1%) in the 600 μg group and 36/55 (65.5%) in the 900 μg cohort; the reasons for discontinuation were unsatisfactory therapeutic effect (*n* = 26; 25.0%), AEs (*n* = 20; 19.2%), subject withdrew consent (*n* = 14; 13.5%), abnormal laboratory value(s), death, condition no longer required study drug, and loss to follow-up (all *n* = 1; 1%).

### Treatment Exposure

Median (range) duration of pasireotide sc exposure from initiation was 25.1 weeks (1–256.4); 82 (78.8%) patients received treatment for ≥12 weeks, 55 (52.9%) were treated for ≥24 weeks, and 36 (34.6%) were treated for ≥48 weeks. Median (range) exposure to pasireotide in all patients who discontinued treatment prematurely (*N* = 64) was 20.7 weeks (1.3–204.0) and in those who discontinued pasireotide prematurely because of AEs (*N* = 20) was 6.9 weeks (1.3–77.1).

Patients received a mean (SD) dose intensity of 1421.0 μg/day (390.1). Mean (SD) dose intensity in all patients who discontinued treatment prematurely was 1442.4 μg/day (380.3) and in those who discontinued treatment prematurely because of AEs was 1239.4 μg/day (428.5). Sixty-six (63.5%) patients had a change in dose intensity, and 28 (26.9%) patients required a dose interruption; AEs/laboratory abnormalities were the most common reason for a change in dose intensity (*n* = 22; 21.2%) and dose interruption (*n* = 23; 22.1%). Pasireotide exposure overall and by dose group is reported in Table 2.

**Table 2:** Pasireotide exposure overall and by dose level.

	**Pasireotide 600 μg bid *N =* 49**	**Pasireotide 900 μg bid *N =* 55**	**All patients *N =* 104**
Median duration of exposure, weeks (range)	24.3 (1–185)	26.0 (3–256)	25.1 (1–256)
Duration of exposure category, *n* (%)			
<12 weeks	13 (26.5)	9 (16.4)	22 (21.2)
12– <24 weeks	11 (22.4)	16 (29.1)	27 (26.0)
24– <36 weeks	7 (14.3)	7 (12.7)	14 (13.5)
36– <48 weeks	3 (6.1)	2 (3.6)	5 (4.8)
≥48 weeks	15 (30.6)	21 (38.2)	36 (34.6)
Mean dose intensity, μg/day (SD)	1066.5 (245.3)	1736.8 (145.7)	1421.0 (390.1)
Number of dose changes, n	41	25	66
Mean (SD)	3.6 (3.7)	3.6 (5.2)	3.6 (4.3)
Median (range)	2.0 (1.0–16.0)	2.0 (1.0–25.0)	2.0 (1.0–25.0)
Number of dose interruptions, n	18	10	28
Mean (SD)	1.4 (1.2)	1.8 (1.9)	1.6 (1.5)
Median (range)	1.0 (1.0–6.0)	1.0 (1.0–7.0)	1.0 (1.0–7.0)

### Safety

Overall, most treatment-emergent AEs were mild to moderate in severity, with 42 (40.4%) patients experiencing drug-related grade 3/4 AEs or drug-related SAEs (primary endpoint), 26 (53.1%) in the 600 μg group and 16 (29.1%) in the 900 μg cohort; the most common by system organ class were metabolism/nutritional (*n* = 20; 19.2%) and gastrointestinal (*n* = 13, 12.5%) disorders, and by preferred term were diabetes mellitus (*n* = 12; 11.5%) and hyperglycemia (*n* = 8; 7.7%) (Table 3).

**Table 3:** Patients who had drug-related grade 3/4 AEs or drug-related SAEs (>1% in all patients) by preferred term and dose group.

**Preferred term**	**Pasireotide 600 μg bid *N =* 49 *n* (%)**	**Pasireotide 900 μg bid *N =* 55 *n* (%)**	**All patients *N =* 104 *n* (%)**
Total	26 (53.1)	16 (29.1)	42 (40.4)
Diabetes mellitus	6 (12.2)	6 (10.9)	12 (11.5)
Hyperglycemia	7 (14.3)	1 (1.8)	8 (7.7)
Diarrhea	4 (8.2)	1 (1.8)	5 (4.8)
Nausea	3 (6.1)	2 (3.6)	5 (4.8)
Blood cortisol decreased	2 (4.1)	1 (1.8)	3 (2.9)
Cholecystitis acute	0	3 (5.5)	3 (2.9)
Adrenal insufficiency	2 (4.1)	0	2 (1.9)

All patients had at least one treatment-emergent AE, regardless of relationship to drug, of which diarrhea (*n* = 53; 51.0%), nausea (*n* = 48; 46.2%), hyperglycemia (*n* = 42; 40.4%), headache (*n* = 31; 29.8%), cholelithiasis (*n* = 30; 28.8%), diabetes mellitus (*n* = 25; 24.0%) and fatigue (*n* = 23; 22.1%) were the most common (≥20% in all patients). Furthermore, most patients (*n* = 102; 98.1%) had at least one AE considered to be drug related (Table 4), with grade 3/4 drug-related AEs observed in 41 (39.4%) patients, the most frequent being diabetes mellitus (*n* = 11; 10.6%) and hyperglycemia (*n* = 8; 7.7%).

**Table 4:** Patients who had drug-related AEs (>10% in all patients) by preferred term and dose group.

**Preferred term**	**Pasireotide 600 μg bid *N =* 49 *n* (%)**		**Pasireotide 900 μg bid *N =* 55 *n* (%)**		**All patients *N =* 104 *n* (%)**	
	**All grades**	**Grade 3/4**	**All grades**	**Grade 3/4**	**All grades**	**Grade 3/4**
Total	48 (98.0)	26 (53.1)	54 (98.2)	15 (27.3)	102 (98.1)	41 (39.4)
Diarrhea	23 (46.9)	4 (8.2)	21 (38.2)	1 (1.8)	44 (42.3)	5 (4.8)
Nausea	18 (36.7)	3 (6.1)	23 (41.8)	2 (3.6)	41 (39.4)	5 (4.8)
Hyperglycemia	18 (36.7)	7 (14.3)	22 (40.0)	1 (1.8)	40 (38.5)	8 (7.7)
Cholelithiasis	7 (14.3)	0	17 (30.9)	1 (1.8)	24 (23.1)	1 (1.0)
Diabetes mellitus	10 (20.4)	6 (12.2)	14 (25.5)	5 (9.1)	24 (23.1)	11 (10.6)
Abdominal pain	9 (18.4)	1 (2.0)	6 (10.9)	0	15 (14.4)	1 (1.0)
Fatigue	8 (16.3)	1 (2.0)	6 (10.9)	0	14 (13.5)	1 (1.0)
Headache	5 (10.2)	1 (2.0)	8 (14.5)	0	13 (12.5)	1 (1.0)
Blood glucose increased	3 (6.1)	0	9 (16.4)	0	12 (11.5)	0

Overall, 30 (28.8%) patients had treatment-emergent SAEs, regardless of relationship to drug, with acute cholecystitis, diabetes mellitus and diarrhea the most common (all *n* = 3; 2.9%). SAEs considered drug related were observed in 16 (15.4%) patients, 10 (20.4%) in the 600 μg group and six (10.9%) in the 900 μg cohort.

Twenty (19.2%) patients discontinued treatment because of AEs (600 μg, *n* = 12 [24.5%]; 900 μg, *n* = 8 [14.5%]). Only six patients discontinued because of hyperglycemia-related AEs (grade 2, *n* = 3; grade 3, *n* = 2; grade 4, *n* = 1), the most common AEs leading to treatment withdrawal: five (10.2%) in the 600 μg cohort and one (1.8%) in the 900 μg group. Gastrointestinal disorders also led to treatment withdrawal in six (5.8%) patients (600 μg, *n* = 5 [10.2%]; 900 μg, *n* = 1 [1.8%]), with abdominal pain and diarrhea the most common (both *n* = 2; 1.9%).

Hyperglycemia-related AEs were documented in 79 (76.0%) patients overall, with the incidence of these AEs similar between dose groups (600 μg, *n* = 35 [71.4%]; 900 μg, *n* = 44 [80.0%)]. The majority of hyperglycemia-related AEs were suspected to be drug related (*n* = 75; 72.1%), and most were of mild-to-moderate severity, with 19 (18.3%) patients experiencing these AEs at grade 3/4 severity (600 μg, *n* = 13 [26.5%]; 900 μg, *n* = 6 [10.9%]) and six (5.8%) patients reporting these events as SAEs. Of the six patients with hyperglycemia-related SAEs, two had the events resolve with dose adjustment/interruption and three discontinued treatment. There were no instances of diabetic ketoacidosis or hyperosmolar hyperglycemia. Most patients with hyperglycemia-related AEs had a medical history of obesity, dyslipidemia, increased blood glucose, hyperinsulinemia and/or diabetes mellitus. At baseline, 73/104 (70.2%) and 63/104 (60.6%) patients, respectively, had fasting plasma glucose (FPG) and HbA_1c_ levels in the normal range (FPG <100 mg/dL; HbA_1c_ <5.7%). Of these patients, last post-baseline FPG and HbA_1c_ levels were in the diabetic range (FPG ≥126 mg/dL; HbA_1c_ ≥6.5%) in 25/73 (34.2%) and 19/63 (30.2%) patients. Of patients with prediabetic levels of FPG (100– <126 mg/dL; *n* = 24/104 [23.1%]) and HbA_1c_ (5.7– <6.5%; *n* = 21/104 [20.2%]) at baseline, 16/24 (66.7%) and 17/21 (80.9%), respectively, had last post-baseline levels in the diabetic range. Overall, 61/104 (58.7%) and 51/104 (49.0%) patients, respectively, had last post-baseline FPG and HbA_1c_ levels within the normal/prediabetic range. Shifts in FPG and HbA_1c_ levels from baseline to last post-baseline value according to diabetic status are shown in Table 5.

**Table 5:** Shift in FPG and HbA_1c_ levels from baseline to last post-baseline value according to diabetic status.

**Baseline value**		**Last post-baseline value**		
**FPG**	***n* (%)**	** <100 mg/dL (normal) *n* (%)**	**100– <126 mg/dL (prediabetic) *n* (%)**	**≥126 mg/dL (diabetic) *n* (%)**
<100 mg/dL (normal)	73 (70.2)	33 (31.7)	15 (14.4)	25 (24.0)
100– <126 mg/dL (prediabetic)	24 (23.1)	3 (2.9)	5 (4.8)	16 (15.4)
≥126 mg/dL (diabetic)	7 (6.7)	3 (2.9)	2 (1.9)	2 (1.9)
Total	104 (100)	39 (37.5)	22 (21.2)	43 (41.3)
**HbA**_**1c**_	***n*** **(%)**	** <5.7% (normal)** ***n*** **(%)**	**5.7– <6.5% (prediabetic)** ***n*** **(%)**	**≥6.5% (diabetic)** ***n*** **(%)**
<5.7% (normal)	63 (60.6)	13 (12.5)	31 (29.8)	19 (18.3)
5.7– <6.5% (prediabetic)	21 (20.2)	0	3 (2.9)	17 (16.3)
≥6.5% (diabetic)	20 (19.2)	0	4 (3.8)	16 (15.4)
Total	104 (100)	13 (12.5)	38 (36.5)	52 (50)

*One patient did not have a last post-baseline HbA_1c_ measurement*.

Gallbladder- and biliary-related AEs were documented in 32 (30.8%) patients, with cholelithiasis (*n* = 30; 28.8%) the most common (Table 6); none of these AEs led to treatment discontinuation. One patient required surgery for an AE of cholelithiasis. Other AEs of special interest included hypocortisolism-related AEs, recorded in eight (7.7%) patients (Table 6). In five patients for whom additional information was provided by the investigator, nine hypocortisolism-related AEs were reported: adrenal insufficiency (*n* = 4 [grade 2, *n* = 1; grade 3, *n* = 3], blood cortisol decreased (*n* = 3 [all grade 3], glucocorticoid deficiency (*n* = 1 [grade 3] and cortisol free urine decreased (*n* = 1 [grade 2]. Of the nine hypercortisolism-related AEs, six had resolved at the patient's last assessment, three after reduction of the pasireotide dose, two after initiation of glucocorticoid supplementation and reduction/interruption of the pasireotide dose, and one after discontinuation of pasireotide treatment.

**Table 6:** Patients with gallbladder- and biliary-, hyperglycemia-, and hypocortisolism-related adverse events, regardless of relationship to drug, by preferred term and maximum grade, overall and by dose group.

**Preferred term**	**Pasireotide 600 μg bid *N =* 49 *n* (%)**		**Pasireotide 900 μg bid *N =* 55 *n* (%)**		**All patients *N =* 104 n (%)**	
	**All grades**	**Grade 3/4**	**All grades**	**Grade 3/4**	**All grades**	**Grade 3/4**
Gallbladder and biliary related	10 (20.4)	0	22 (40.0)	3 (5.5)	32 (30.8)	3 (2.9)
Cholelithiasis	8 (16.3)	0	22 (40.0)	1 (1.8)	30 (28.8)	1 (1.0)
Cholecystitis acute	0	0	3 (5.5)	3 (5.5)	3 (2.9)	3 (2.9)
Biliary dilatation	1 (2.0)	0	1 (1.8)	0	2 (1.9)	0
Bile duct stone	0	0	1 (1.8)	1 (1.8)	1 (1.0)	1 (1.0)
Cholecystitis	0	0	1 (1.8)	0	1 (1.0)	0
Cholecystitis chronic	0	0	1 (1.8)	0	1 (1.0)	0
Gallbladder disorder	1 (2.0)	0	0	0	1 (1.0)	0
Hyperbilirubinemia	1 (2.0)	0	0	0	1 (1.0)	0
Hyperglycemia related	35 (71.4)	14 (28.6)	44 (80.0)	7 (12.7)	79 (76.0)	21 (20.2)
Hyperglycemia	20 (40.8)	8 (16.3)	22 (40.0)	1 (1.8)	42 (40.4)	9 (8.7)
Diabetes mellitus	10 (20.4)	6 (12.2)	15 (27.3)	6 (10.9)	25 (24.0)	12 (11.5)
Blood glucose increased	4 (8.2)	0	9 (16.4)	0	13 (12.5)	0
Type 2 diabetes	5 (10.2)	1 (2.0)	3 (5.5)	0	8 (7.7)	1 (1.0)
HbA_1c_ increased	1 (2.0)	0	1 (1.8)	0	2 (1.9)	0
Impaired fasting glucose	1 (2.0)	0	1 (1.8)	0	2 (1.9)	0
Blood insulin decreased	0	0	1 (1.8)	0	1 (1.0)	0
Glucose tolerance impaired	1 (2.0)	0	0	0	1 (1.0)	0
Hypocortisolism related	7 (14.3)	4 (8.2)	1 (1.8)	1 (1.8)	8 (7.7)	5 (4.8)
Adrenal insufficiency	4 (8.2)	2 (4.1)	1 (1.8)	0	5 (4.8)	2 (1.9)
Blood cortisol decreased	3 (6.1)	2 (4.1)	1 (1.8)	1 (1.8)	4 (3.8)	3 (2.9)
Cortisol free urine decreased	2 (4.1)	0	0	0	2 (1.9)	0
Glucocorticoid deficiency	1 (2.0)	1 (2.0)	0	0	1 (1.0)	1 (1.0)

Most patients experienced an AE that required additional therapy (*n* = 91; 87.5%), with 81 (77.9%) patients requiring additional therapy to manage an AE that was suspected to be related to pasireotide treatment (Table 7); hyperglycemia was the most common AE requiring additional therapy (*n* = 31; 29.8%). Various glucose-lowering drugs were used for the management of hyperglycemia, including metformin (*n* = 40, 38.5%), metformin hydrochloride (*n* = 12, 11.5%), dipeptidyl peptidase-4 (DPP-4) inhibitors (*n* = 20, 19.2%; primarily vildagliptin [*n* = 7, 6.7%] and sitagliptin phosphate [*n* = 6, 5.8%]), sulfonylureas (*n* = 20, 19.2%; primarily gliclazide [*n* = 8, 7.7%] and glimepiride [*n* = 7, 6.7%]), and insulin (*n* = 30, 28.8%). One patient died during the study, with the death attributed to a ‘natural cause' and not considered related to study treatment.

**Table 7:** Patients with adverse events requiring additional therapy (>2% all patients), regardless of relationship to drug, by preferred term and maximum grade, overall and by dose group.

**Preferred term**	**Pasireotide 600 μg bid *N =* 49 *n* (%)**		**Pasireotide 900 μg bid *N =* 55 *n* (%)**		**All patients *N =* 104 *n* (%)**	
	**All grades**	**Grade 3/4**	**All grades**	**Grade 3/4**	**All grades**	**Grade 3/4**
Total	44 (89.8)	29 (59.2)	47 (85.5)	18 (32.7)	91 (87.5)	47 (45.2)
Hyperglycemia	14 (28.6)	8 (16.3)	17 (30.9)	1 (1.8)	31 (29.8)	9 (8.7)
Diabetes mellitus	10 (20.4)	6 (12.2)	13 (23.6)	6 (10.9)	23 (22.1)	12 (11.5)
Nausea	5 (10.2)	3 (6.1)	10 (18.2)	2 (3.6)	15 (14.4)	5 (4.8)
Diarrhea	8 (16.3)	3 (6.1)	5 (9.1)	0	13 (12.5)	3 (2.9)
Headache	6 (12.2)	1 (2.0)	7 (12.7)	1 (1.8)	13 (12.5)	2 (1.9)
Nasopharyngitis	2 (4.1)	0	7 (12.7)	0	9 (8.7)	0
Blood glucose increased	1 (2.0)	0	6 (10.9)	0	7 (6.7)	0
Type 2 diabetes mellitus	3 (6.1)	1 (2.0)	3 (5.5)	0	6 (5.8)	1 (1.0)
Hypertension	3 (6.1)	2 (4.1)	3 (5.5)	1 (1.8)	6 (5.8)	3 (2.9)
Abdominal pain	4 (8.2)	1 (2.0)	2 (3.6)	0	6 (5.8)	1 (1.0)
Hypokalemia	1 (2.0)	1 (2.0)	4 (7.3)	2 (3.6)	5 (4.8)	3 (2.9)
Hypercholesterolemia	1 (2.0)	0	3 (5.5)	0	4 (3.8)	0
Back pain	1 (2.0)	0	3 (5.5)	1 (1.8)	4 (3.8)	1 (1.0)
Depression	2 (4.1)	1 (2.0)	2 (3.6)	0	4 (3.8)	1 (1.0)
Gastroenteritis	2 (4.1)	0	2 (3.6)	0	4 (3.8)	0
Urinary tract infection	0	0	4 (7.3)	0	4 (3.8)	0
Fatigue	3 (6.1)	1 (2.0)	1 (1.8)	0	4 (3.8)	1 (1.0)
Edema peripheral	2 (4.1)	1 (2.0)	2 (3.6)	0	4 (3.8)	1 (1.0)

### Efficacy

**Figure 1:**
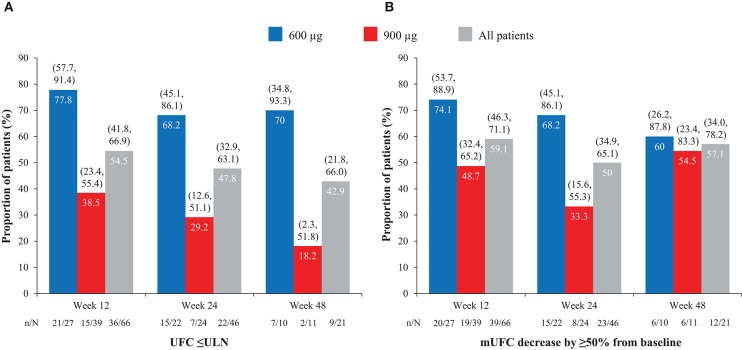
Proportion of patients with **(A)** normalized mUFC and **(B)** a decrease in mUFC of ≥50% from baseline at weeks 12, 24, and 48, overall and by dose group. Values in parentheses are two-sided 95% exact CIs.

Overall, of patients with evaluable UFC measurements at weeks 12, 24, and 48, respectively, 36/66 (54.5%), 22/46 (47.8%), and 9/21 (42.9%) had mUFC ≤ULN at those time points; 39/66 (59.1%), 23/46 (50.0%), and 12/21 (57.1%) patients had a reduction in mUFC of ≥50% from baseline at these time points (Figure 1). After imputation of last available post-baseline mUFC assessments for patients who had discontinued/missing values, 34/86 (39.5%) and 30/86 (34.9%) patients had mUFC ≤ULN at weeks 24 and 48, respectively. Mean (95% CI) percentage change in mUFC from baseline to weeks 12, 24, and 48 is shown in Table 8.

**Table 8:** Mean change in mUFC from baseline to weeks 12, 24, and 48 by dose group.

	**600 μg bid *N =* 49**		**900 μg bid *N =* 55**		**All patients *N =* 104**	
	**Mean, nmol/24 h**	**Mean change from baseline, % (95% CI)**	**Mean, nmol/24 h**	**Mean change from baseline, % (95% CI)**	**Mean, nmol/24 h**	**Mean change from baseline, % (95% CI)**
Baseline	853.5	–	752.0	–	799.8	–
Week 12	120.9	−60.0 (−71.8, −48.3)	291.2	−34.1 (−49.0, −19.2)	221.6	−44.7 (−54.9, −34.5)
Week 24	113.5	−58.8 (−72.4, −45.3)	444.2	−24.1 (−42.2, −6.1)	286.0	−40.7 (−52.6, −28.8)
Week 48	108.8	−60.2 (−77.1, −43.2)	518.5	−23.1 (−64.3, 18.2)	323.4	−40.7 (−63.3, −18.2)

**Figure 2:**
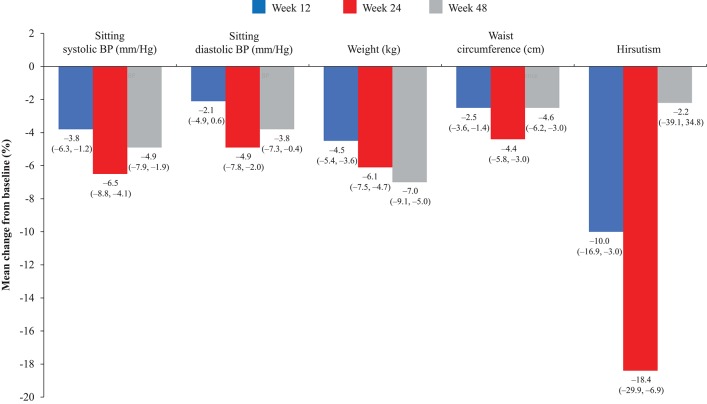
Mean percentage change in signs and symptoms of hypercortisolism from baseline to weeks 12, 24, and 48 in the overall study population. Values in parentheses are two-sided 95% exact CIs.

**Figure 3:**
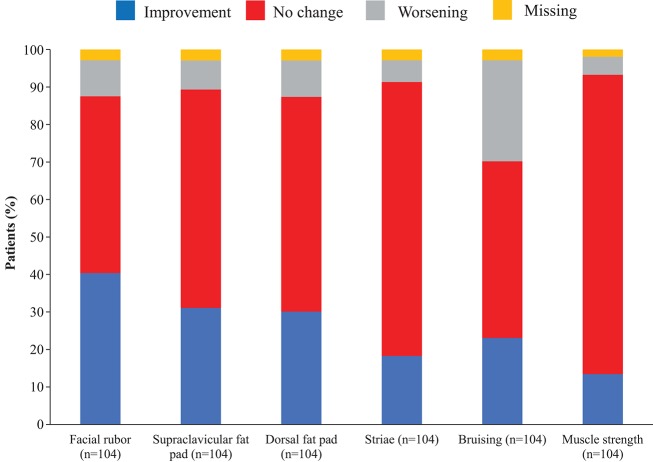
Proportion of patients with improvement, no change or worsening of categorical signs of Cushing's disease from baseline to last post-baseline value in the overall study population.

**Figure 4:**
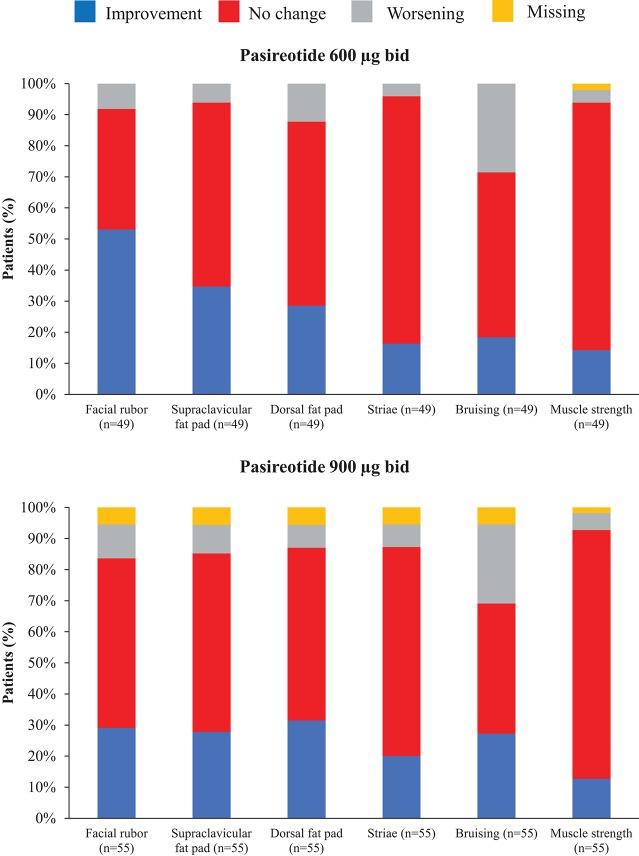
Proportion of patients with improvement, no change or worsening of categorical signs of Cushing's disease from baseline to last post-baseline value by dose group.

Overall, continuous signs and symptoms (BP, weight, waist circumference, and hirsutism) were improved at week 12, with improvements sustained through to week 48 (Figure 2). A favorable shift in all studied categorical signs of Cushing's disease (facial rubor, striae, bruising, muscle strength, supraclavicular, and dorsal fat pads) was observed from baseline to last post-baseline assessment overall (Figure 3) and by dose group (Figure 4). QoL also improved, as indicated by the mean (95% CI) percentage increase from baseline in CushingQoL score of 67.1% (30.0, 104.3) at week 12, 82.3% (25.2, 139.4) at week 24, and 34.4% (19.5, 49.4) at week 48. Changes in clinical signs and symptoms and HRQoL overall and by dose group are reported in Table 9.

**Table 9:** Mean change from baseline to weeks 12, 24, and 48 in continuous signs/symptoms of hypercortisolism, and quality of life, overall and by dose group.

**Clinical parameter**	**Pasireotide 600 μg bid *N =* 49 Mean, % (95% CI)**	**Pasireotide 900 μg bid *N =* 55 Mean, % (95% CI)**	**All patients *N =* 104 Mean, % (95% CI)**
**WEEK 12**			
Sitting SBP, mmHg	−6.8 (−10.3, −3.4)	−1.4 (−5.0, 2.3)	−3.8 (−6.3, −1.2)
Sitting DBP, mmHg	−4.6 (−8.9, −0.2)	−0.2 (−3.8, 3.4)	−2.1 (−4.9, 0.6)
Weight, kg	−4.2 (−5.2, −3.1)	−4.8 (−6.2, −3.4)	−4.5 (−5.4, −3.6)
Waist circumference, cm	−2.0 (−3.2, −0.7)	−2.9 (−4.7, −1.2)	−2.5 (−3.4, −1.4)
Hirsutism (females only), Ferriman–Gallway score	−12.2 (−20.9, −3.7)	−8.2 (−19.0, 2.6)	−10.0 (−16.9, −3.0)
QoL, CushingQoL score	21.3 (7.4, 35.2)	100.8 (37.6, 164.0)	67.1 (30.0, 104.3)
**WEEK 24**			
Sitting SBP, mmHg	−7.4 (−10.6, −4.3)	−5.7 (−9.2, −2.2)	−6.5 (−8.8, −4.1)
Sitting DBP, mmHg	−6.2 (−8.8, −3.7)	−3.8 (−8.8, 1.2)	−4.9 (−7.8, −2.0)
Weight, kg	−7.3 (−9.2, −5.4)	−5.2 (−7.2, −3.1)	−6.1 (−7.5, −4.7)
Waist circumference, cm	−5.8 (−8.0, −3.6)	−3.1 (−4.9, −1.7)	−4.4 (−5.8, −3.0)
Hirsutism (females only), Ferriman–Gallway score	−21.2 (−35.5, −6.8)	−16.2 (−34.4, 2.0)	−18.4 (−29.9, −6.9)
QoL, CushingQoL score	36.7 (15.5, 57.9)	119.7 (16.2, 223.2)	82.3 (25.2, 139.4)
**WEEK 48**			
Sitting SBP, mmHg	−5.1 (−8.9, −1.4)	−4.7 (−9.5, −0.0)	−4.9 (−7.9, −1.9)
Sitting DBP, mmHg	−3.3 (−7.8, 1.3)	−4.3 (−9.5, 1.0)	−3.8 (−7.3, −0.4)
Weight, kg	−8.0 (−11.0, −5.0)	−6.3 (−9.3, −3.3)	−7.0 (−9.1, −5.0)
Waist circumference, cm	−5.1 (−7.6, −2.6)	−4.1 (−6.3, −2.0)	−4.6 (−6.2, −3.0)
Hirsutism (females only), Ferriman–Gallway score	−18.2 (−34.7, −1.7)	9.6 (−55.8, 75.0)	−2.2 (−39.1, 34.8)
QoL, CushingQoL score	24.0 (6.8, 41.1)	42.3 (18.8, 65.8)	34.4 (19.5, 49.4)

*DBP, diastolic blood pressure; SBP, systolic blood pressure*.

## Discussion

The favorable efficacy and safety profile of pasireotide sc in patients with Cushing's disease has previously been established in a Phase III trial (16). This expanded-access study confirms that pasireotide sc is similarly tolerated in a large population of patients with Cushing's disease in an international, real-world, clinical-practice setting. Over the course of the study, 40% of patients experienced drug-related grade 3/4 or SAEs, the primary endpoint of the study. All patients experienced at least one AE during the study, regardless of relationship to pasireotide, with the most frequent related to gastrointestinal discomfort (reported in ~83% of patients). Although common, most AEs were manageable, with <20% of patients discontinuing treatment for this reason.

Hyperglycemia-related AEs were documented in 76% of patients, consistent with previous reports of pasireotide sc (16). Notably, few patients reported clinically significant hyperglycemia-related AEs, with grade 3/4 or SAEs of hyperglycemia and diabetes mellitus experienced by 8 and 12% of patients, respectively; there were no instances of diabetic ketoacidosis or hyperosmolar hyperglycemia observed in the study. Most hyperglycemia-related AEs were manageable, and <6% of patients discontinued treatment because of these events. There was no strict protocol for the management of hyperglycemia-related AEs, owing to the observational nature of the study. However, in line with expert recommendations (24), which suggest stepwise management of pasireotide-associated hyperglycemia with metformin followed by the sequential addition of a DPP-4 inhibitor, glucagon-like peptide 1 (GLP-1) agonist and insulin, metformin (39% of patients) and DPP-4 inhibitors (19% of patients) were the most frequently used antidiabetic medications in this study. Most patients (79%) completed ≥12 weeks (≥3 months) of treatment, which, given that most glucose elevations typically manifest soon after pasireotide initiation (regardless of baseline glucose status) and peak after 1 month of treatment (16, 25, 26), suggests that the majority of patients remained on treatment long enough for appropriate treatment decisions to be made regarding management of pasireotide-associated hyperglycemia. Furthermore, glucose levels have been reported to stabilize after their initial rise (25), with the rate and severity of hyperglycemia shown to be mostly unchanged after its initial onset for up to 5 years' treatment with pasireotide sc (26). Impairments in glucose metabolism are common in patients with Cushing's disease, which result from glucocorticoid-related stimulation of gluconeogenesis, as well as increased insulin resistance in the liver and skeletal muscles (4). In this study, 30 and 39% of patients, respectively, had FPG and HbA_1c_ levels in the prediabetic/diabetic range at baseline (FPG ≥100 mg/dL; HbA_1c_ ≥5.7%). It is noteworthy that 59% and 49% of patients had a last post-baseline FPG and HbA_1c_ level, respectively, within the normal/prediabetic range (FPG <126 mg/dL; HbA_1c_ <6.5%), and that most patients who reported hyperglycemia-related AEs had prior history of related illness before pasireotide initiation (namely, glucose intolerance or diabetes mellitus, conditions associated with a predisposition to the occurrence of hyperglycemia-related AEs during pasireotide treatment) (25). Furthermore, most patients with normal glucose tolerance at baseline did not have FPG and HbA_1c_ levels in the diabetic range at last post-baseline assessment. This would suggest that control of glucose levels prior to initiation of pasireotide may reduce the likelihood of hyperglycemia occurring during pasireotide treatment. Blood glucose levels need to be closely monitored in patients receiving pasireotide, particularly in patients with pre-existing glucose-related disorders, and glucose-lowering therapy should be initiated as necessary (24). An ongoing Phase IV study is currently investigating the optimal management of pasireotide-associated hyperglycemia (clinicaltrials.gov, identifier: NCT02060383).

Treatment with somatostatin analogs is commonly associated with gallstone formation, although this rarely is symptomatic or requires surgery (16, 27). In our study, 29% of patients had cholelithiasis. Notably, none of these AEs required discontinuation of pasireotide, and only one necessitated surgical intervention. Gallbladder ultrasound is advised at regular intervals during pasireotide treatment (5), and cholecystectomy should be considered for symptomatic patients.

Our analysis shows that 48 and 43% of evaluable patients had normalized mUFC at weeks 24 (6 months) and 48 (12 months), respectively. It should be noted that many patients did not have evaluable mUFC measurements at all time points, and that a contributing factor to this was the stipulation in the study protocol that patients only received pasireotide as part of the study until the drug became commercially available in their respective countries. Considering normalization of mUFC in this study after imputation of last available mUFC assessments for patients who had discontinued/missing values, 40 and 35% of patients had mUFC ≤ULN at weeks 24 and 48, respectively. In a Phase III study, pasireotide sc normalized UFC levels in 22 and 19% of patients (including those who received a dose increase after 3 months' treatment) with Cushing's disease after 6 and 12 months' treatment, respectively (16). As such, results from our real-world study support those shown in a clinical trial setting, demonstrating that pasireotide is effective at normalizing mUFC levels in patients with Cushing's disease. Results from this real-world study are not directly comparable to those from the Phase III clinical trial because of differences in study design and analysis. Importantly, while the primary aim of the Phase III trial was to assess the efficacy of two doses of pasireotide sc (600 and 900 μg) after 6 months of treatment in patients with Cushing's disease, the main purpose of our study was to provide additional safety data on the use of pasireotide sc in a real-world setting. It is also important to note that it is not possible to directly compare mUFC response rates between studies. Unlike in the Phase III trial, which assessed response according to the intention-to-treat principle, the real-world nature of our study mandated that assessment of response was based on the number of patients who had evaluable UFC measurements at each time point. The benefits of our study lie within its observational nature. Real-world studies complement those performed in highly controlled trial settings by providing information on treatment practices and patient characteristics of populations typically excluded from clinical trials. Moreover, real-world studies are useful for identifying less frequent side effects of medications and for helping to guide treatment decisions.

In this study, the proportion of patients reported to have a normalized mUFC level at each time point was lower in the 900 μg cohort than in the 600 μg group. While it is important to note that this study was neither designed nor statistically powered to assess differences between dose cohorts, one possible explanation for the apparent difference in mUFC response rates between dose groups is that patients in the 600 μg cohort may have had a lesser degree of hypercortisolism at baseline, as evidenced by the lower baseline mUFC level of this group compared with the 900 μg group. Indeed, clinical trials of pasireotide have shown that normalization of mUFC is more likely to be achieved in patients with lower baseline/screening mUFC levels (16, 28). Furthermore, normalization of UFC is likely influenced by patients being able to receive a dose increase during the study. Therefore, the differences between dose groups in UFC normalization at time points should be interpreted with caution. Clinical signs and symptoms, as well as HRQoL, were also improved in our analysis, with the extent of change observed similar to that previously reported for pasireotide sc (16, 29). Reductions in systolic and diastolic BP, waist circumference and weight, and improvements in QoL, were observed by week 12, with improvements sustained through to week 48, indicating continued therapeutic benefit during long-term treatment with pasireotide sc. Indeed, HRQoL impairment is a long-term multifactorial physical and psychosocial issue for patients with Cushing's disease that is known to persist even after achieving disease control (30). Although improvements in certain signs and symptoms, namely hirsutism, appeared to be dampened at week 48 compared with prior time points, this is likely related to the smaller number of women with evaluable measurements at this time compared with weeks 12 and 24.

Patients treated with pasireotide have been reported to derive significant clinical benefit even without normalization of UFC. In a subanalysis of the Phase III study of pasireotide sc in patients with Cushing's disease, patients without full control of UFC levels exhibited significant improvements in BP, weight, body mass index and waist circumference; notably, improvements in BP were greatest in patients who did not receive antihypertensive medications (29). Such patients may be suitable candidates for combination therapy.

Few studies have evaluated the efficacy and safety of pasireotide in real-world clinical practice. In the studies carried out in this setting to date, the results have supported the findings from prospective studies. In one study conducted in five Italian centers, 68% of patients with mild–moderate disease (*N* = 31) achieved a response to pasireotide (defined as normalization or near normalization of UFC [<1.0–1.1 × ULN]) after 6 months of treatment, which was accompanied by improvements in clinical signs and symptoms (19). The safety profile of pasireotide was consistent with that previously observed during the Phase III study of pasireotide in Cushing's disease (16), with hyperglycemia a frequent (81% of patients) but manageable side effect (study goal of HbA_1c_ <7.5% achieved in 65% of patients). The efficacy of pasireotide has also been demonstrated in a small, 12-month, single-center study; all five patients who received pasireotide were biochemically controlled after 1 year of treatment, with four of these patients also maintaining normal glycemic control (17). The long-term efficacy of pasireotide has been demonstrated in another single-center study: 50% of patients (*N* = 20) treated with pasireotide sc for a median (range) of 9 (3–72) months had normalized mUFC at their last follow-up (18). Finally, another Italian multicenter study highlighted the potential cardioprotective effects of pasireotide treatment, with 12 months of treatment leading to significant reductions in cardiometabolic risk factors (20). While these real-world studies provide key insights into the use of pasireotide in clinical practice, they are limited by their small patient populations.

Several of the currently available medical therapies have demonstrated efficacy for the treatment of Cushing's disease in retrospective studies. In one study of 200 patients treated with ketoconazole, 49.0% of patients were reported to have attained control of UFC levels at last follow-up (31). Tolerability issues led to treatment withdrawal in 20.5% of patients, with hepatotoxicity (18.5% of patients reported increased liver enzyme levels) the main safety issue concerning this agent, which is reversible for some patients following treatment discontinuation (32). In a retrospective analysis of 195 patients with Cushing's disease, ~64% of 38 patients treated with metyrapone achieved normalized UFC levels, with treatment generally well-tolerated and discontinuation (permanent or temporary) because of AEs reported in 23% of patients (33). Mifepristone has been shown to moderate the metabolic burden of Cushing's disease patients, as well as induce positive changes in clinical signs and symptoms, in an open-label, 24-week trial (34). Although adrenal insufficiency is a potential serious side effect of mifepristone treatment (35), it was rarely reported in the 24-week trial. Despite these agents showing efficacy in some patients with Cushing's disease, it is imperative that selection of medical therapy is tailored to the individual clinical situation (11), and for the treatment selected, careful monitoring for relevant AEs should be performed, with appropriate action taken should any arise. Several combinations of medical therapies have been used to treat patients with Cushing's disease, mostly in small retrospective studies (8, 12, 15). One prospective study to date demonstrated how the addition of cabergoline and ketoconazole to pasireotide in a stepwise approach in patients biochemically uncontrolled on pasireotide alone increased the number of patients with normalized mUFC (36).

While the primary endpoint of this expanded-access study was to document the safety and tolerability of pasireotide sc, the analysis of efficacy was confounded by the nature of the study, whereby patients only received pasireotide as part of the study until the drug became commercially available in their respective countries, precluding evaluation of UFC normalization in many patients.

In conclusion, this is the first study to our knowledge to report detailed safety of pasireotide sc in a large, international population of patients with Cushing's disease in clinical practice. The findings from this study provide valuable insights into the use of pasireotide in the real world; pasireotide sc was generally tolerated, with no new safety signals identified. While hyperglycemia-related AEs were frequent, most were manageable, with <6% of patients discontinuing because of these events. The efficacy data presented here demonstrate the effectiveness of pasireotide sc in real-world clinical practice.

## Data Availability

Novartis is committed to sharing with qualified external researchers access to patient-level data and supporting clinical documents from eligible studies. These requests are reviewed and approved by an independent review panel on the basis of scientific merit. All data provided are anonymized to respect the privacy of patients who have participated in the trial, in line with applicable laws and regulations. This trial data availability is done according to the criteria and processes described at www.clinicalstudydatarequest.com.

## Ethics Statement

This study was carried out in accordance with the recommendations of the Declaration of Helsinki with written informed consent from all subjects. All subjects gave written informed consent in accordance with the Declaration of Helsinki. The protocol was approved by the independent ethics committee or institutional review board for each participating center (Supplementary Appendix).

## Author's Note

The study was designed by the academic investigators and the sponsor, Novartis Pharma AG. Data were collected by investigators using Novartis' data management systems and analyzed by Novartis' statistical team. The academic investigators enrolled patients in the study. Writing, reviewing, amending of the manuscript the first draft was prepared by a medical writer funded by Novartis Pharmaceuticals Corporation.

## Author Contributions

All authors contributed to data interpretation. All the authors made the decision to submit the manuscript for publication and vouch for the accuracy and completeness of the data. FC performed the statistical and data analysis.

### Conflict of Interest Statement

MF has received grants to her university as a clinical investigator for Novartis, Millendo and Strongbridge Biopharma, as well as occasional scientific consulting fees from Novartis and Strongbridge Biopharma. CI is employed by Panda Medical Associates. LS and TM have received personal fees as clinical investigators for Novartis. RM and FC are full-time employees of Novartis Pharmaceuticals Corporation. The authors declare that this study received funding from Novartis. The funder designed the study with the academic investigators and analyzed the data, which were collected by the investigators using Novartis's data management systems. Writing, reviewing, and amending of the manuscript's first draft was carried out by a medical writer funded by Novartis Pharmaceuticals Corporation. The remaining author declares that the research was conducted in the absence of any commercial or financial relationships that could be construed as a potential conflict of interest.
