# Supplementary material for: Safety and Efficacy of Subcutaneous Pasireotide in Patients With Cushing's Disease: Results From an Open-Label, Multicenter, Single-Arm, Multinational, Expanded-Access Study
Source: Front Endocrinol (Lausanne). 2019 Jul 16;10:436. doi: 10.3389/fendo.2019.00436 (PMC6646464; doi:10.3389/fendo.2019.00436)
Supplement: Supplementary file 1 [file Data_Sheet_1.pdf]

Safety and efficacy of subcutaneous pasireotide in patients with Cushing's disease: Results from an open-label, multicenter, single-arm, multinational, expanded-access study

Maria Fleseriu, Chioma Iweha, Luiz Salgado, Tania Longo Mazzuco, Federico Campigotto, Ricardo Maamari, Padiporn Limumpornpetch

Supplementary Appendix

List of Independent Ethics Committees or Institutional Review Boards that provided ethics approval for the study

Institutional Review Board for Human Research at St. Joseph's Hospital and Medical Center (Phoenix, Arizona), Quorum Review Institutional Review Board (Seattle, Washington), University of Pennsylvania (Philadelphia, Pennsylvania), University of California Los Angeles (Los Angeles, California), Oregon Health & Science University (Portland, Oregon), Mount Sinai School of Medicine (New York, New York), Vanderbilt University (Nashville, Tennessee), Cook County Health & Hospitals System (Chicago, Illinois), Western Institutional Review Board (Olympia, Washington), University of Nebraska Medical Center (Omaha, Nebraska), Comite de Etica em Pesquisa em Seres Humanos do Instituto de Assistencia Medica ao Servidos Publico Estadual (Sau Paulo, Brazil), CAPPesq - Comissao de Etica para Analise de Projetos de Pesquisa do Hospital das Clinicas da Faculdade de Medicina da USP (Sau Paulo, Brazil), Comite de Etica em Pesquisa do Hospital Municipal Sao Jose (Joinville, Brazil), Comite de Etica Em Pesquisas – HGF (Fortaleza, Brazil), CEP do(a) Irmandade da Santa Casa de Londrina (Londrina, Brazil), Comite de Etica em Pesquisa da Associacao Hospitalar Moinhos de Vento (Porto Alegre Rio Grande do Sul, Brazil), Kyung Hee University Hospital Instituional Review Board (Seoul, Korea), Samsung Medical Center Insitutional Review Board (Seoul, Korea), Severance Hospital, Yonsei University Health System (Seoul, Korea), Seoul National University College of Medicin/Seoul National University Hospital Institutional Review Board (Seoul, Korea), Institutional Review Board Faculty of Medicine Chulalongkorn University (Bangkok, Thailand), Human Research Ethics Committee, Faculty of Medicine, Prince of Songkla University (Had Yai Songkia, Thaliand), Scientific Council of AHEPA University Hospital (Thessaloniki, Greece), Scientific Council of General Hospital of Thessaloniki "Ippokrateio" (Thessaloniki, Greece), Scientific Council of General Hospital of Athens "G. Gennimatas" (Athens, Greece), Scientific Council of Evangelismos Athens General Hospital (Athens, Greece), Comite Etico de Investigacion Clinica del Hospital Universitario i Politecnico La Fe (Valencia, Spain), Comite Etico de Investigacion Clinica del Consorcio Hospital General Universitario de Valencia (Valencia, Spain), Comite d'Etica de la Investigacio Hospital Universitari Germans Trias I Pujol (Badalona, Spain), Comite Etico de Investigacion Clinica Hospital Universitario Reina Sofia (Cordoba, Spain), Comite Etico de Investigacion de Granada (Granada, Spain), Comite Etico de Investigacion Clinica de Galicia (Santigo de Compostela, Spain), Comisia Nationala de Bioetica a Medicamentului si a Dispozitivelor Medicale (Bucharest, Romania), Eticka komise Vseobecne fakultni nemocnice v Praze (Prague, Czech Republic), MONIKI n.a. Vladimirskiy (Moscow, Russia), Ludwig-Maximilians Universitat Munchen Medizinische Fakultat Ethikkommission (Munich, Germany), Landesamt fur Gesundheit und soziales Geschaftsstelle der Ethik-Kommission des

Landes Berlin (Berlin, Germany), Friedrich-Alexander Universitat Erlangen-Numberg Medizinische Fakultat Ethik-Kommission (Erlangen, Germany), Ethik-Kommission bei der Medizinischen Fakultat der Universitat Wurzburg Institut fur Pharmakologie und Toxikologie (Wurzburg, Germany), Ethik-Kommission des Fachbereichs Medizin der Johann Wolfgang Goethe-Universitat (Frankfurt, Germany), Bayerische Landesarztekammer Ethik-Komission (Munich, Germany), Arztekammer Hamburg Geschaftsstelle der Ethik-Kommission (Hamburg, Germany), Ethikkommission der Universitatmedizin Gottingen (Gottingen, Germany), Ethik-Kommission an der Medizinischen Fakultat der RWTH Aachen Universitatsklinikum Aachen (Aachen, Germany), Landesarztekammer Rheinland-Pfalz Ethik-Kommission (Mainz, Germany), Comite d ethique, Hotel Dieu (Beirut, Lebanon)
